# Dynamic changes in body composition during XELOX/SOX chemotherapy in patients with gastric cancer

**DOI:** 10.3389/fonc.2024.1309681

**Published:** 2024-04-30

**Authors:** Zhen-Hao Li, Ting Xu, Ya-Juan Zhang, Jing-Hang Jiang, Yu-Ze Mi, Jia-Xuan Li, Jing Shen, Yi-Rui Fu, Bo-Ying Qin, Fan Lin, Dong-Jing Fu, Mei-Jin Yue, Shu-Mei Ma, Quan-Fu Li

**Affiliations:** ^1^ The Clinical class 6 of First Clinical Medical College, Wenzhou Medical University, Wenzhou, China; ^2^ Department of Medical Oncology, Ordos Central Hospital, Ordos, China; ^3^ School of Optometry & Ophthalmology, Wenzhou Medical University, Wenzhou, China; ^4^ Ordos Clinical College, Inner Mongolia Medical University, Ordos, China; ^5^ Ordos Clinical College, Baotou Medical College, Ordos, China; ^6^ School of Public Health and Management, Wenzhou Medical University, Wenzhou, China

**Keywords:** dynamic changes, gastric cancer, skeletal muscle, XELOX/SOX, neutropenia

## Abstract

**Objectives:**

In this study, we compared the dynamic changes in body composition during XELOX/SOX chemotherapy in patients with gastric cancer. Furthermore, we investigated the potential impact of these changes on the occurrence of toxic side effects.

**Methods:**

Patients with gastric cancer who received adjuvant or first-line XELOX/SOX chemotherapy between January 2020 and June 2023 were enrolled. The Brief Conghua Scale was used to assess energy intake, and nutritional management was carried out with reference to the *Chinese Guidelines for Nutritional Therapy of Cancer 2020*. The NRS 2002 Nutritional Risk Screening Scale, PG-SGA scale, bioelectrical impedance analysis, and dynamic changes in lumbar 3 vertebral skeletal muscle index were compared between baseline and post-chemotherapy in the study. The neutropenia was evaluated using the *Common Terminology Criteria for Adverse Events V.5.0*, developed by the National Institutes of Health.

**Results:**

Dynamic follow-up was completed in 39 cases, with a mean follow-up time of 117.62 ± 43.38 days. The incidence of sarcopenia increased significantly after chemotherapy, escalating from 46.2% to 51.3%. After chemotherapy, the mean L3SMI decreased from 36.00 cm^2^/m^2^ to 34.99 cm^2^/m^2^. Furthermore, when compared to pre-chemotherapy values, the body composition indexes body mass index (BMI), SL3, fat mass free index (FFMI), lean body mass (LBM), and body surface area (BSA) were significantly reduced after chemotherapy. Regardless of baseline or post-chemotherapy status, the incidence of grade ≥ 3 neutropenia was significantly higher in the sarcopenia group than in the non-sarcopenia group. Furthermore, when the skeletal muscle index decreased during chemotherapy, the incidence of grade ≥ 3 neutropenia was significantly higher in both the sarcopenia and non-sarcopenia groups compared to baseline. When the incidence of grade ≥ 3 neutropenia in the post-chemotherapy sarcopenia group was compared to baseline status, the increase was significantly higher in the sarcopenia group than in the maintenance/increase group.

**Conclusions:**

Skeletal muscle mass decreased progressively during XELOX/SOX chemotherapy in gastric cancer patients, followed by a higher incidence of grade ≥ 3 neutropenia.

## 1 Introduction

According to the Global Cancer Statistics 2020 (GLOBOCAN), gastric cancer has the fifth highest incidence rate and the fourth highest mortality rate among all cancers globally. There are significant geographical variations in its incidence and mortality rates. Gastric cancer ranks third in China in terms of both incidence and mortality rates (1). XELOX (capecitabine + oxaliplatin) or SOX (tegafur, gimeracil, and oteracil potassium + oxaliplatin) is the recommended chemotherapy regimen for postoperative adjuvant or advanced first-line treatment in patients with gastric cancer (2, 3). Chemotherapy is associated with a progressive loss of skeletal muscle mass, and the mechanisms involved include uncontrolled muscle protein catabolism, chemotherapy-induced mitochondrial damage, an increase in reactive oxygen species, an increase in tumor growth factor (TGF) β protein, and a reduction in muscle microvasculature, among others. Adverse events during chemotherapy such as fatigue, loss of appetite, nausea, vomiting, and diarrhea negatively affect food intake and physical activity and further lead to muscle quantity and quality decreasing dramatically (4).

Due to their specific physiological functions, malignant tumors of the digestive system have a significant impact on human nutritional status. Clinical investigations have confirmed that the L3 skeletal muscle index (L3SMI) decreases significantly in patients with gastric cancer during chemotherapy. Meta-analyses have also revealed that patients with gastric cancer with sarcopenia have increased chemotherapy-related toxicities and a poor prognosis (5, 6). Skeletal muscle mass is currently assessed using bioelectrical impedance analysis (BIA), dual-energy x-ray absorptiometry (DXA), computed tomography (CT) and magnetic resonance imaging (MRI). Among them, L3SMI has been shown to correlate well with whole-body muscle. Due to the prevalence of CT examinations in patients with tumors, it is widely recognized as the gold standard for measuring muscle mass, particularly in patients with tumors (7, 8).

Our research team has concentrated on the dynamic monitoring of sarcopenia. According to published reviews, skeletal muscle loss can occur during antitumor therapy. In the meanwhile, past research has shown that active nutritional interventions can help patients with gastrointestinal cancer with sarcopenia maintain anabolic muscle metabolism potential, prevent muscle loss and even gain muscle (9, 10). However, current data in this field, are primarily based on retrospective analyses of European and American populations or clinical investigations that include a variety of gastrointestinal tumors (9, 10).

Thus, a prospective, self-controlled clinical study was conducted in the context of real-world clinical nutritional management to analyze the dynamic changes in body composition during chemotherapy in gastric cancer patients receiving XELOX/SOX and assess the impact on toxic side effects.

## 2 Materials and methods

A prospective study was conducted to investigate the dynamic changes in L3SMI in patients with gastric cancer admitted to the Department of Medical Oncology of Ordos Central Hospital between January 2020 and June 2023. In a self-controlled study, the patients underwent adjuvant or first-line six-cycle XELOX/SOX chemotherapy. The study was approved by the Medical Ethics Committee of Ordos Central Hospital (2021-013). All participants provided written informed consent, and the study was registered with the China Clinical Trial Center (ChiCTR2200056758).

### 2.1 Enrollment criteria

#### 2.1.1 Inclusion criteria

① Age ≥ 18 years old, pathologically confirmed gastric cancer to receive SOX or XELOX regimen chemotherapy, oxaliplatin dosage strength of 130 mg/m^2^, tegafur, gimeracil, and oteracil potassium of 40 mg/m^2^, or capecitabine of 1000 mg/m^2^; ② Expected survival time ≥ 6 months which was evaluated by clinical doctors based on imaging examination and laboratory testing results, besides these evaluation also considering ZPS Performance Status and PG-SGA scale; ③ No abnormalities in liver and kidney function, blood routine, electrocardiogram prior to chemotherapy, blood test must satisfy the following requirements: leukocytes > 3.5 × 10^9/^L, neutrophils > 1.5 × 10^9^/L, platelets > 85 × 10^9^/L, alkaline phosphatase ≤ 2.5 times the upper limit of normal, serum alanine transferase (ALT) and aspartate aminotransferase (AST) ≤ 3 times the upper limit of normal, bilirubin ≤ 1.5 times the upper limit of normal, creatinine ≤ 1.5 times the upper limit of normal; ④ No other contraindications to chemotherapy; ⑤ CT scan and the initiation of chemotherapy without resorting to interventions such as surgery to alter body composition; ⑥ Individuals who can undergo regular follow-up assessments using BIA and CT; ⑦ Those who are capable of providing informed consent and signing the consent form.

#### 2.1.2 Exclusion criteria

① patients with hypersensitivity to oxaliplatin, tegafur, gimeracil, and oteracil potassium, or capecitabine; ② patients receiving prophylactic application of long-acting drug for increasing white cells after chemotherapy; ③ Patients who were bedridden at the time of enrollment and could not cooperate with nutritional assessment; ④ those with other serious consumptive diseases, such as diabetes; ⑤ those with serious mental illnesses and poor compliance; ⑥ those who discontinued antitumor therapy on their own or did not have regular follow-up with body composition analysis or side effects; ⑦ those who were deemed unsuitable for enrollment in the study by the investigator.

### 2.2 Clinical nutritional management measures

The energy intake of enrolled patients with gastric cancer was evaluated using the Concise Conghua Scale which have been published on our team previously reported clinical nutrition research paper although the method is not validated in GI tumors patients (9), and clinical nutritional management was carried out during hospitalization or outpatient visits using methods recommended by the *China Oncology Nutritional Guidelines* of the Committee on Oncology Nutrition and Supportive Therapies of the China Anti-Cancer Association (11, 12). The daily intake of the patients (including dietary, enteral, and/or parenteral nutrition) must be at least 70% of the target value. The protein requirement was 1.0~2.0 g/kg/d, which was calculated using the optimum body weight of the individual. When the next step fails to meet 60% of the target requirement for more than 3~5 days, the nutritional treatment plan from the previous step is selected. The assessment results and the patient’s preferences were used to determine whether tube-fed enteral nutrition or parenteral nutrition support should be provided.

## 3 Data collection

### 3.1 Information collection

After the initial admission, general information such as name, age, pathological stage, tumor site, and adjuvant or first-line chemotherapy regimen was collected.

### 3.2 Height and weight measurements

After each admission, patients underwent height and weight measurements using standard methods. The following formulas were used to compute BMI and BSA: BMI = weight (kg)/height^2 (m^2^); BSA (m^2^) = [height (cm) + weight (kg) - 60]/100.

### 3.3 Body composition measurements

① NRS 2002 and PG-SGA assessment: Enrolled patients were evaluated on a scale by a full-time nutritional nurse specializing in oncology within 12 hours after hospital admission; NRS ≥ 3 was classified as being at nutritional risk. The PG-SGA assessment yielded the following results: A/≤ 1 (well-nourished); B/2-8 (suspected or moderate malnutrition); and C/≥ 9 (severe malnutrition);

② BIA test: Using a clinical nutritional testing analyzer (model: CNA-H1), all enrolled patients underwent body composition measurements, including body fat, skeletal muscle mass, and body fat percentage. When measuring body composition using the bioelectrical impedance method, it was necessary to maintain a suitable ambient temperature (20°C–25°C), measure on an empty stomach, empty the bowels before the measurement, and avoid measuring after exercise, showering, or during menstruation for females. Use the following formula to compute the Fat-Free Mass Index (FFMI): (1- percentage of body fat) × weight (kg) ÷ height (m^2^). The FFMI low standard is ≤ 17.4 kg/m^2^ for males and ≤ 15.0 kg/m^2^ for females (12).

③ Abdominal CT scanning: Abdominal CT lumbar 3 vertebrae consecutive level scanning without contrast was performed within 30 days of chemotherapy medication after patient enrollment, -70 to +150 HU units, with 5 mm as layer thickness, and two consecutive transverse level images of the same sequence of the lumbar 3 vertebrae were selected to analyze the muscle area, and radiation therapy TPS software (XIO) was used to calculate the skeletal muscles (including the psoas major muscle, erector spinae, quadratus lumborum, transversus abdominis muscles, external abdominal obliques, and internal abdominal obliques) by summing their cross-sectional areas. L3 skeletal muscle index (SMI) = L3 vertebral skeletal muscle area (cm²)/height² (cm²) (13). LBM (whole body lean body soft mass) = 0.3 * [L3 vertebral skeletal muscle area (cm^2^)] + 6.06 (13).

### 3.4 Laboratory tests and toxic side effects

The fasting venous blood of patients was drawn and sent for examination within 24 hours of drug administration, and the indexes, including liver function, renal function, and peripheral blood hemogram, were recorded. Hematologic and non-hematologic toxicity were dynamically monitored during the inter-chemotherapy period, and the toxic side effects of neutropenia were graded and recorded using CTCAE5.0 (14).

## 4 Statistical methods

RStudio 4.3.1 software and SPSS 26.0 statistical software were used to analyze the data. A baseline-corrected linear model was used to analyze the data from before and after changes to each index. Measurements conforming to the normal distribution are expressed as the mean ± standard deviation (`x ± s). If they did not conform to the normal distribution, they were analyzed using the Wilcoxon signed-rank sum test and the Mann-Whitney U test. Frequency-distributed count data are expressed as rates. The paired-sample t-test was used to analyze the data for each index before and after changes. The Pearson’s test and the chi-squared test were used to conduct correlation analysis, with *P* < 0.05 indicating a statistically significant difference.

## 5 Results

### 5.1 Clinical characteristics of the patients

The study comprised 65 patients; however, only 39 were able to complete the dynamic follow-up due to the influence of the COVID-19 pandemic. **Table 1** shows the clinical characteristics of the patients with gastric malignancy who participated in the study. According to **Table 1**, the majority of the patients (84.6%) were male. The average age of the patients was 60.21 ± 11.17 years, and the duration of follow-up was 117.62 ± 43.38 days. According to the evaluation criteria for Asian patients with tumors with sarcopenia, male patients were classified as sarcopenic if their L3SMI was < 36 cm^2^/m^2^, while female patients were classified as sarcopenic if their L3SMI was < 29 cm^2^/m^2^ (15). Based on these parameters, all patients were divided into two groups: the sarcopenia group (group A) and the non-sarcopenia group (group B). In addition, the BMI grouping method used in this study was based on the criteria outlined in the *Chinese Guidelines for Nutritional Therapy of Cancer 2020* (12). According to the guidelines, 1) a BMI less than 18.5 was considered indicative of underweight or malnutrition; 2) a BMI between 18.5 and 23.9 was considered normal; and 3) a BMI between 24 and 27.9 was considered overweight. The prevalence of sarcopenia in the study population was found to be 46.2% (18/39). Nutritional management was offered to patients in a clinical setting, with 89.7% of patients receiving nutritional education and parenteral nutritional support, while 10.3% received gastrointestinal nutritional tube support.

**Table 1:** Clinical characteristics of patients.

Characteristics	Sarcopenia	Non-Sarcopenia	Total
	(N=18) (A)	(N=21) (B)	(N=39)
Age, Year	62.44 ± 7.58	58.29 ± 13.41	60.21 ± 11.17
Follow-up time, Day	110.67 ± 45.84	123.57 ± 41.35	117.62 ± 43.38
Gender			
Man	16 (88.9%)	17 (81.0%)	33 (84.6%)
Female	2 (11.1%)	4 (19.0%)	6 (15.4%)
Tumour location			
Preventriculus	9 (50.0%)	6 (28.6%)	15 (38.5%)
Gastric body	2 (11.1%)	8 (38.1%)	10 (25.6%)
Pylorus	7 (38.7%)	7 (33.3%)	14 (35.7%)
Type of chemotherapy			
Adjuvant chemotherapy	11 (61.1%)	18 (85.7%)	29 (74.4%)
Palliative chemotherapy	7 (38.9%)	3 (14.3%)	10 (25.6%)
Nutritional Intervention			
Nutritional tube	4 (22.2%)	0 (0%)	4 (10.3%)
Nutrition education	14 (77.8%)	21 (100%)	35 (89.7%)
Body mass index (kg/m^2^)			
<18.5 Underweight	8 (44.4%)	5 (23.8%)	13 (33.3%)
18.5-23.9 Normal	9 (50.0%)	12 (57.1%)	21 (53.9%)
24.0-27.9 Overweight	1 (5.6%)	4 (19.1%)	5 (12.8%)

### 5.2 Alterations in body composition-related indicators before and after chemotherapy

The patients were divided into two groups: the baseline chemotherapy group (G1 group) and the end-of-chemotherapy group (G2 group). **Table 2** summarizes the alterations observed in BMI, SL3, L3SMI, LBM, FFMI, weight, BSA, PG-SGA Scale, and NRS-2002 Scale, as well as grade ≥ 3 neutropenia in all patients before and after chemotherapy. Within the framework of clinical nutritional management in the real world, the PG-SGA Nutritional Assessment Scale and NRS-2002 Scale exhibited a significant decrease following chemotherapy as compared to the pre-chemotherapy values (*P* = 0.000, *P* = 0.000). However, the body composition indexes, including BMI, SL3, FFMI, LBM, and BSA, exhibited statistically significant decreases in their disparities following chemotherapy compared to pre-chemotherapy levels (*P* = 0.000, *P* = 0.0457, *P* = 0.01586, *P* = 0.0458, *P* = 0.0457). L3SMI also exhibited a decrease, but it was not statistically significant.

**Table 2:** Comparison of body composition related indicators between G1 and G2.

	G1	G2	Percent Change (%)	*P*
BMI (kg/m^2^)	21.11 ± 3.42	20.48 ± 3.14	-2.59 ± 6.87	0.000
<18.5	17.37 ± 1.03	17.43 ± 0.72	0.011 ± 0.068	0.6058
18.5-23.9	21.24 ± 1.53	21.09 ± 1.33	-0.41 ± 0.066	0.0066
24.0-27.9	26.39 ± 1.50	26.60 ± 1.51	-0.041 ± 0.083	0.437
SL3 (cm^2^)	103.27 ± 25.64	99.51 ± 25.20	-1.17 ± 17.98	0.0457
L3SMI (cm^2^/m^2^)	36.00 ± 7.73	34.99 ± 7.83	-2.60 ± 11.50	0.1615
LBM (kg)	37.04 ± 7.69	35.91 ± 7.56	-2.72 ± 9.38	0.0458
FFMI (kg/m^2^)	17.04 ± 1.76	17.29 ± 1.63	1.66 ± 5.00	0.01586
PG-SGA	8.13 (6.89,9.37)	5.72 (4.67,6.77)		0.000
NRS 2002	4.15 (3.43,4.88)	3.00 (2.33.3.67)		0.000

BMI, body mass index; SL3, skeletal muscle area of the third lumbar spine.

L3SMI, L3 skeletal muscle index; LBM, lean body mass; FFMI, fat-free mass index.

BSA, body surface area.

**Figure 1:**
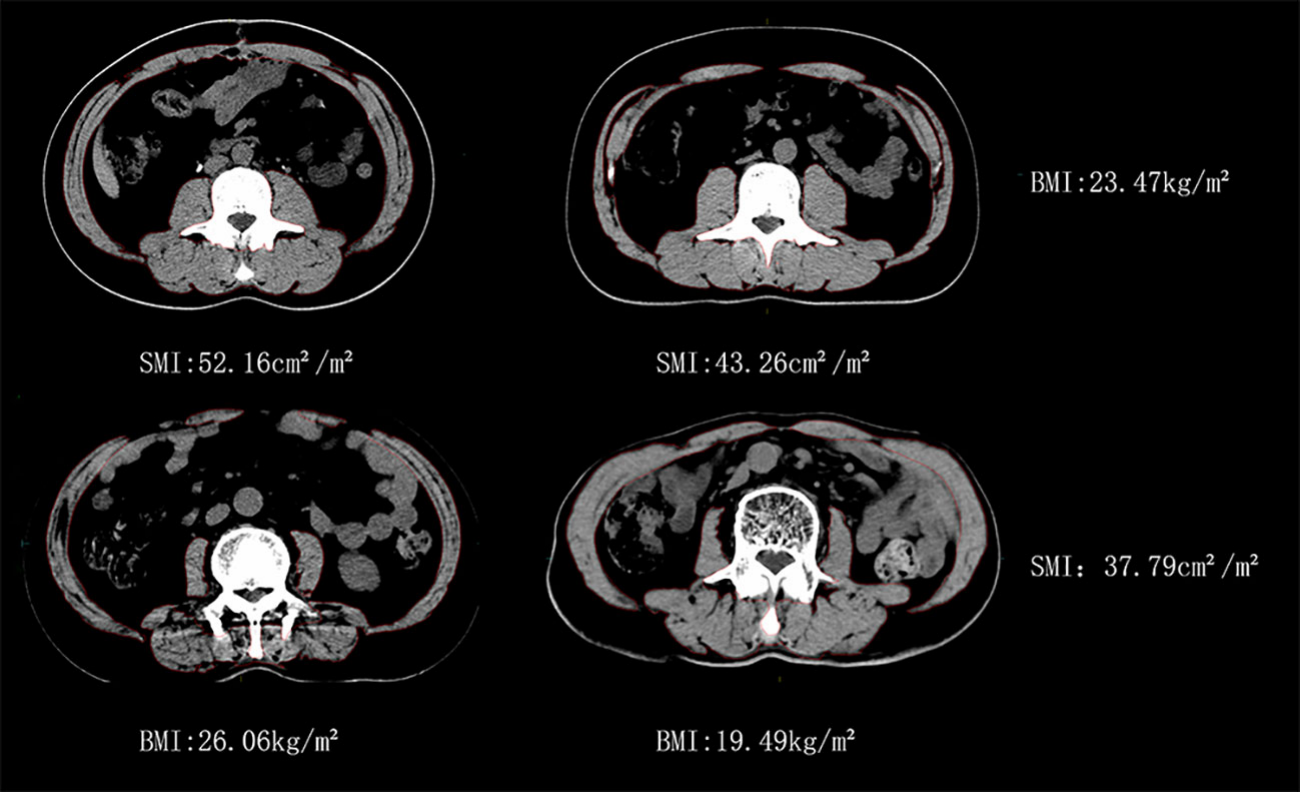
Similar BMI with different SMI vs similar SMI with different BMI for man patients.

Individuals had significant variations in BMI and SMI, as depicted in **Figure 1**. Specifically, two male patients, C1 and C2, exhibited identical BMI values, yet their SMIs differed by 17.1%. Similarly, two male patients, D1 and D2, displayed the same SMI, but their BMIs differed by 25.1%.

### 5.3 Dynamic fluctuations in SMI before and after chemotherapy

**Table 3** shows a comparative analysis of the dynamic changes in L3SMI before and after chemotherapy in 39 patients. The patients were divided into two groups: the baseline chemotherapy group (Group G1) and the end-of-chemotherapy group (Group G2). Patients experienced a reduction in L3SMI at the end of chemotherapy when compared to the baseline chemotherapy group, regardless of gender, adjuvant chemotherapy, or nutrient tube placement. The L3SMI increased by 0.1 ± 2.64 cm^2^/m^2^ after the end of palliative chemotherapy compared to baseline chemotherapy. Similarly, those who received nutritional education experienced an increase of 0.1 ± 2.90 cm^2^/m^2^ in L3SMI at the end of chemotherapy as compared to baseline chemotherapy.

**Table 3:** Change in L3SMI according to different clinical characteristics (cm^2^/m^2^).

	G1	G2	Percentage Decrease (%)	Absolute value Decrease	*P*
Gender					
Man	35.39 ± 7.54	34.33 ± 7.74	0.275 ± 0.125	1.06 ± 4.25	0.138
Female	39.35 ± 8.63	38.54 ± 8.01	0.174 ± 0.029	0.82 ± 1.14	0.124
Tumour location					
Preventriculus	32.61 ± 7.23	30.74 ± 7.77	0.053 ± 0.159	1.87 ± 4.69	0.201
Gastric body	39.97 ± 8.25	38.17 ± 7.12	0.042 ± 0.098	1.81 ± 4.61	0.212
Pylorus	36.78 ± 6.73	37.24 ± 6.71	-0.014 ± 0.044	-0.4 ± 1.74	0.362
Type of Chemotherapy					
Adjuvant Chemotherapy	36.65 ± 8.14	35.21 ± 8.43	0.385 ± 0.122	1.44 ± 4.24	0.079
Palliative Chemotherapy	34.12 ± 6.37	34.30 ± 6.06	-0.010 ± 0.085	-0.1 ± 2.64	0.827
NutritionalIntervention					
Nutritional tube	36.50 ± 7.56	35.34 ± 7.89	0.031 ± 0.117	1.16 ± 4.03	0.906
Nutrition education	31.61 ± 8.94	31.79 ± 7.42	-0.018 ± 0.104	-0.1 ± 2.90	0.099

### 5.4 Alterations in skeletal muscle parameters

**Figure 2:**
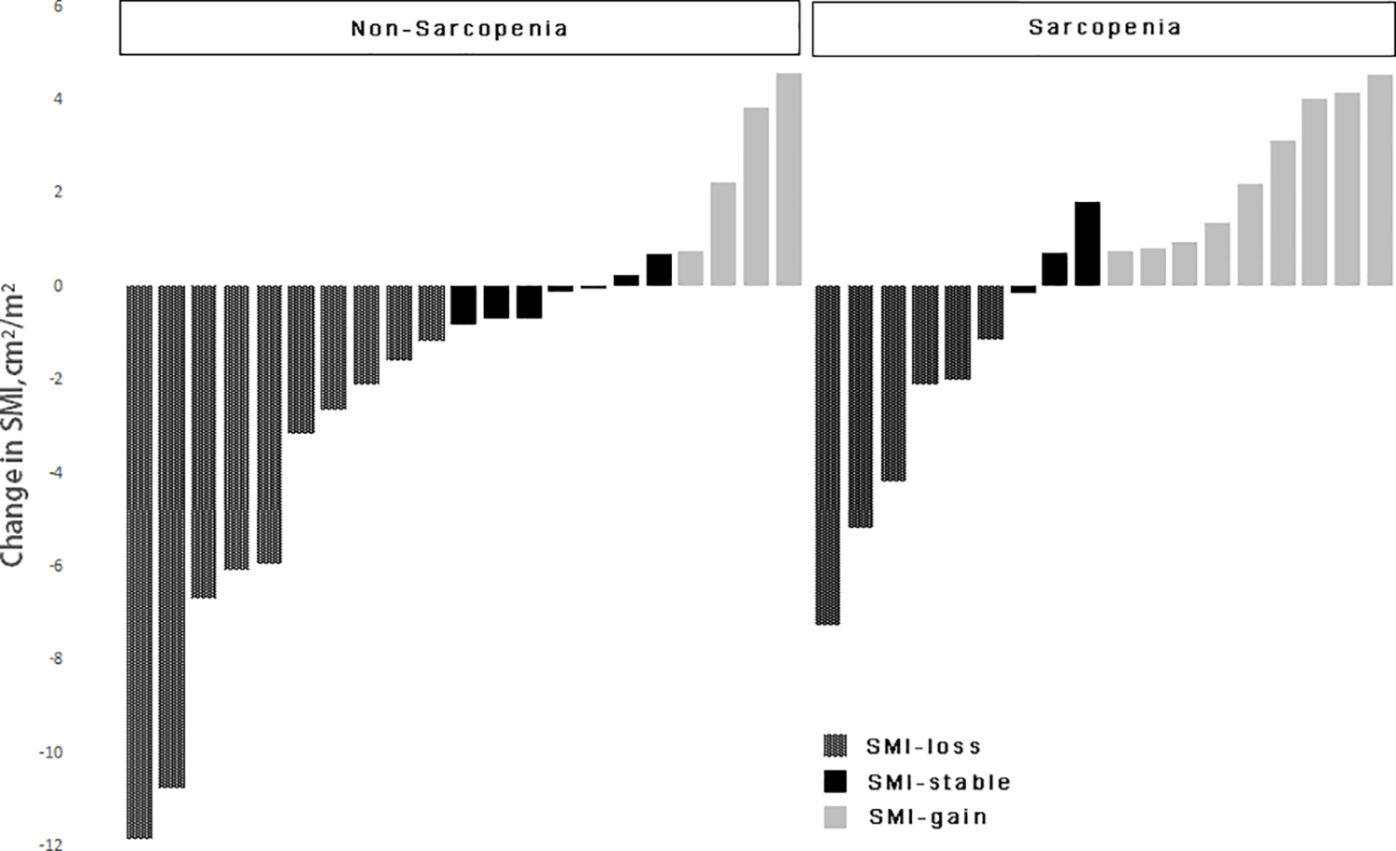
Change in skeletal muscle index in patients according to sarcopenia.

Based on the change in SMI before and after chemotherapy, patients were divided into three groups. The first group consisted of patients who had skeletal muscle loss, defined as a decrease in SMI of more than 2%. Patients in the second group had an increase in skeletal muscle, defined as an increase in SMI of more than 2%. The third group included patients whose SMI remained stable, with changes that fell within the range in the two previous groups (16). Based on the previously described sarcopenia thresholds (male: L3SMI < 36 cm^2^/m^2^, female: L3SMI < 29 cm^2^/m^2^), a total of 39 patients were classified into sarcopenia and non-sarcopenia groups based on their SMI values prior to the initiation of chemotherapy, as illustrated in **Figure 2**. The prevalence of sarcopenia increased significantly, escalating from 46.2% prior to chemotherapy to 51.3% subsequent to chemotherapy. The mean SMI of the entire cohort exhibited a decrease from 36.00 cm^2^/m^2^ prior to chemotherapy to 34.99 cm^2^/m^2^ post-chemotherapy. This reduction corresponded to an average decrease of 1.68 kg of LBM and a decrease of 1.17 cm^2^ in the skeletal muscle area of the lumbar 3 vertebral region. SMI remained stable or increased during the course of chemotherapy in 23 out of 39 (59.0%) of the patients who were enrolled in the research. The SMI increased by 1.49 cm^2^/m^2^ on average (95% CI: 0.72-2.25 cm^2^/m^2^, *P* = 0.001). SMI, on the other hand, decreased in 16 out of 39 (41.0%) of the patients. The mean decrease in SMI was 4.62 cm^2^/m^2^ (95% CI: 2.97–6.27 cm^2^/m^2^, *P* < 0.001). In the cohort of patients who did not exhibit sarcopenia before chemotherapy, 52.4% (11/21) demonstrated either stable or increased muscle mass. In contrast, 66.7% (12/18) of patients with sarcopenia exhibited the same trend (*P* = 0.062).

### 5.5 Sarcopenia during chemotherapy and its association with chemotherapy-related toxicities

Thirty-nine patients were divided into two groups based on the presence or absence of sarcopenia: the sarcopenia group and the non-sarcopenia group. Its relationship between the initial chemotherapy and the incidence of grade ≥ 3 toxic side effects during chemotherapy was examined and presented in **Table 4**. The incidence of grade ≥ 3 neutropenia in the baseline sarcopenia group was shown to be statistically significant when compared to the non-sarcopenia group (*P* = 0.0004). After chemotherapy, there was a statistically significant increase in the occurrence of grade ≥ 3 neutropenia in patients with sarcopenia compared to those without sarcopenia (*P* = 0.0024).

**Table 4:** Relevance between sarcopenia and toxicity reactions.

	Sarcopenia (n=18)	Non-Sarcopenia (n=21)	*P*
Baseline status	8 (44.4%)	0 (0.0%)	0.0004
Whole process	15 (83.3%)	8 (38.1%)	0.0024

### 5.6 Changes in SMI during chemotherapy as well as chemotherapy-related toxic side effects

A total of 39 patients were divided into two groups based on their change in SMI, either maintained, increased or lost. **Table 5** shows the results of an analysis of the relationship between their baseline status and the occurrence of grade ≥ 3 or higher neutropenia during chemotherapy follow-up.

**Table 5:** Relevance between SMI change and toxicity reactions.

	SMI stable/gain (n=23)	SMI loss (n=16)	*P*
Baseline status	5 (21.7%)	3 (18.8%)	0.4112
Whole process	12 (52.2%)	11 (68.8%)	0.1534

### 5.7 Categorization of treatment modalities and chemotherapy-related toxicities during chemotherapy

A total of 39 patients were divided into two groups based on their treatment modality: postoperative adjuvant chemotherapy and palliative chemotherapy. The relationship between initial chemotherapy and the occurrence of grade ≥ 3 or more toxic side effects during the course of chemotherapy was investigated. **Table 6** displays the results of this investigation.

**Table 6:** Relevance between type of chemotherapy and toxicity reactions.

	Adjuvant chemotherapy (n=29)	Palliative chemotherapy (n=10)	*P*
Baseline status	3 (10.3%)	5 (50.0%)	0.0041
Whole process	14 (48.3%)	9 (90.0%)	0.0112

## 6 Discussion

On a global scale, gastric cancer continues to be a highly prevalent and fatal form of cancer, with a particular impact on elderly males. The cumulative risk of developing gastric cancer globally, from birth to 74 years of age, is reported to be 1.87% in men and 0.79% in women (17). The National Cancer Center of China published the most recent issue of national cancer statistics in February 2022. It elaborated on the burden of cancer in China in 2016. That year, there were 4,064,000 new cases of cancer, with 396,500 of them being new cases of gastric cancer, which ranked third among all types of cancer (18). Gastrointestinal malignancies have the most significant impact on human nutritional status due to their special physiological functions. Sarcopenia is a condition that causes skeletal muscle mass and function to decline. Recent studies have shown that perioperative sarcopenia in gastric cancer is not only an independent risk factor for postoperative surgical complications (hazard ratio (HR) = 2.330, 95% confidence interval (CI) = 1.132–4.796, *P* = 0.022), but it also has an impact on long-term oncologic outcomes (19–24). Sarcopenia was revealed to be an independent prognostic factor for both overall survival (OS) and cancer-specific survival (CSS) in patients with gastric cancer. [HR (95% CI) for OS: 1.82 (1.32–2.47), *P* < 0.001; HR (95% CI) for CCS: 1.73 (1.02–2.80), *P* = 0.043] (19–24). Huang et al. from Wenzhou Medical University in China examined the diagnostic value of CT measurements of L3SMI in predicting complications and long-term survival following radical gastrectomy for gastric cancer. The results of the study indicated that CT diagnosis of skeletal sarcopenia outperformed nutritional screening techniques in predicting postoperative complications and survival (25).

Our research team conducted dynamic monitoring of sarcopenia using CTL3SMI diagnosis. Previous research has confirmed that active nutritional interventions can help patients with gastrointestinal sarcopenia retain their anabolic muscle metabolism potential, prevent muscle loss, or even gain muscle. However, the patients in this research had a wide range of gastrointestinal tumors, including gastric, pancreatic, and colorectal cancers, and were treated with the same chemotherapy regimen (9, 10). As a result, this prospective clinical study modified the enrollment criteria and chemotherapy regimens. It included 39 gastric cancer patients who underwent adjuvant or first-line 6 cycles of XELOX/SOX chemotherapy and completed dynamic follow-up. The evaluation criteria for sarcopenia in Asian oncology patients were adopted. The following criteria were used in this study: for males, L3SMI < 36 cm^2^/m^2^; for females, L3SMI < 29 cm^2^/m^2^ (15). According to these parameters, the incidence of sarcopenia in the patients enrolled in the current research was 46.2% (18/39). The incidence of sarcopenia in patients receiving adjuvant chemotherapy at the time of radical surgery was significantly higher at 37.9% (11/29) compared to 18.98% (167/880) in a study of operable gastric cancer by Huang et al. Huang et al. used the significantly broader diagnostic criteria recommended by the European Working Group on Sarcopenia in Older People (EWGSOP), which were L3SMI < 40.8 cm^2^/m^2^ for standard males and L3SMI < 34.9 cm^2^/m^2^ for females (25). If the EWGSOP criteria were used to evaluate the patients enrolled in this study, the percentage of patients with sarcopenia would be 66.7% (26/39). Sarcopenia was diagnosed in 25 of the 33 male patients. It is worth noting that even though 25.6% (10/39) of the patients had advanced gastric cancer, the incidence of sarcopenia in patients who underwent adjuvant chemotherapy with radical surgery was as high as 65.5% (19/29). Although the small sample size may affect the results, it also suggests that when using L3SMI criteria to diagnose sarcopenia and conduct related clinical research, it is critical to consider not only the racial differences but also the potential impact of different cancer types and disease stages on the diagnostic criteria for sarcopenia. This indirectly implies that Ordos, a region in western China, has a higher frequency of sarcopenia in patients diagnosed with gastric cancer.

Previous research on the diagnosis of sarcopenia using L3SMI criteria has primarily focused on the correlation between baseline status, toxic side effects of treatment, and prognosis (6). However, there has been an increased emphasis on examining the relationship between changes in skeletal muscle dynamics before and after treatment and their impact on side effects, efficacy, and prognosis since 2015. In fact, after 2018, 60% of the studies examining changes in skeletal muscle dynamics during treatment were published (9). In the current study, we compared the dynamic changes in L3SMI in patients with gastric cancer undergoing XELOX/SOX chemotherapy with their baseline status. It has been observed in real-world clinical practice that there are statistically significant reductions in the NRS 2002 Nutritional Risk Screening Scale and PG-SGA Nutritional Assessment Scale before and after chemotherapy, which can be attributed to nutritional education and other modalities (*P* = 0.000, *P* = 0.000). The risk of developing malnutrition decreased significantly. However, as indicated in **Table 2**, lumbar 3 vertebral muscle area (SL3), lean body soft mass (LBM), and fat free mass index (FFMI) all decreased statistically significantly during chemotherapy,. Although the mean L3SMI, the main observational index of the entire cohort, decreased from 36.00 cm^2^/m^2^ before chemotherapy to 34.99 cm^2^/m^2^ after chemotherapy (*P* = 0.1615), resulting in an average decrease of 1.68 kg in whole-body lean body mass, this decrease did not reach statistical significance. This lack of significance may be attributed to the fact that the 65 enrolled patients were affected by the COVID-19 epidemic, with only 39 completing dynamic follow-up (26). According to the error criteria of 2% for L3SMI measurement using CT, the decrease in L3SMI in this group of patients was -2.60 ± 11.50%. Therefore, the change in skeletal muscle mass values before and after chemotherapy was evaluated as a decrease in this group of patients (16). This is consistent with the findings of Park et al. in a study of 111 patients receiving first-line palliative chemotherapy for advanced gastric cancer. As chemotherapy was administered, the median SMI values decreased by -4.0 cm^2^/m^2^, -4.5 cm^2^/m^2^, and -3.8 cm^2^/m^2^, respectively, in patients with objective remission, stable disease, or progressive disease (5). Similarly, Florian Huemer et al. (2023) reported a statistically significant decrease in body composition parameters during perioperative FLOT chemotherapy for gastric cancer (51.3 vs. 48.8 cm^2^/m^2^, *P* = 0.02) (27). The aforementioned studies investigated the correlation between changes in L3SMI over time and survival rates. In all of these investigations, baseline sarcopenia (*P* = 0.021) and a decrease in SMI during chemotherapy (*P* = 0.032) were significant negative predictors for survival (5, 27). In this investigation, the decrease in L3SMI before and after chemotherapy in patients with gastric cancer (-2.60 ± 11.50%) was similar to the decrease observed during chemotherapy for gastrointestinal tumors in our previous research (-2.05 ± 9.21%) (10). However, it was significantly lower than the decrease in L3SMI observed in advanced gastric cancer patients undergoing palliative chemotherapy by Park et al. in 2020 (-11.30 ± 13.00%) and the decrease in L3SMI observed in advanced gastric cancer patients undergoing palliative chemotherapy reported by Yusuke Yamaoka et al. (2015) in Japan (6.20 ± 6.80%) (27, 28). Different disease stages among enrolled patients, variations in sample sizes, and disparities in baseline thresholds for sarcopenia between populations can all contribute to differences in the extent of L3SMI decline before and after chemotherapy across studies. The implementation of clinical nutritional interventions also played a role in the lower magnitude of L3SMI decline observed in this investigation.

The effect of changes in L3SMI dynamics on chemotherapy-induced neutropenia in patients with gastric cancer following XELOX/SOX chemotherapy was also investigated in this study. Previous research has reported a clear relationship between sarcopenia and chemotherapy toxicities (29). Our study, which was published in 2022, confirmed that patients with sarcopenia who underwent albumin-paclitaxel chemotherapy experienced a significant increase in the incidence of grade ≥ 3 neurotoxicity and neutropenia (30, 31). Data from the current study focused on the relationship between dynamic changes in skeletal muscle index and neutropenia. The results of the study showed that, regardless of baseline status or chemotherapy follow-up, the incidence of grade ≥ 3 neutropenia was significantly higher in the sarcopenia group compared to the non-sarcopenia group (44.4% vs. 0.00%; 83.3% vs. 38.1%). Furthermore, the skeletal muscle index declined significantly with six cycles of chemotherapy in both the sarcopenia and non-sarcopenia groups. In addition, compared to baseline, the prevalence of grade ≥ 3 neutropenia was significantly higher in both groups (83.3% vs. 44.4%; 38.1% vs. 0.00%). The findings are consistent with those reported in the above investigation and in a prospective multicenter study of 51 patients with advanced colorectal cancer conducted by Barrat et al., in which sarcopenia was the sole factor associated with grade 3–4 toxicity (30–32). The prevalence of sarcopenia among the enrolled patients increased from 46.2% before chemotherapy to 51.3% after chemotherapy. **Figure 2** shows that in patients with pre-chemotherapy sarcopenia, the rate of muscle mass stabilization or increase was 66.7% (12/18), which was higher than the rate of 52.4% (11/21) in non-sarcopenic patients. When the incidence of grade ≥ 3 neutropenia after chemotherapy follow-up was compared to baseline status in the muscle loss group, the increase of 50.0% in the muscle loss group was greater than the increase of 30.5% in the maintenance/increase group. The specific values were 68.8% vs. 18.8 and 52.2% vs. 21.7%, respectively. This is consistent with the findings of our prior study on gastrointestinal tumors. It indicates that implementing adequate nutritional interventions may enhance skeletal muscle levels and reduce the occurrence of grade ≥ 3 neutropenia in patients with sarcopenia (10). The incidence of grade ≥ 3 neutropenia was evaluated in patients who underwent adjuvant chemotherapy versus advanced palliative chemotherapy in this study. Both at baseline and after six cycles of chemotherapy, the incidence of grade ≥ 3 neutropenia was significantly lower in patients who received adjuvant chemotherapy (10.3% vs. 50.0%; 48.3% vs. 90.0%). Furthermore, the baseline incidence of sarcopenia was 41.38% (12/29), which was lower than the incidence of 60.0% (6/10) in patients who received advanced palliative chemotherapy. Furthermore, patients who received adjuvant chemotherapy had a higher incidence of non-sarcopenia (58.62%, or 17/29) compared to 40.0% (4/10) in patients who received advanced palliative chemotherapy. The findings also support the association between patients with sarcopenia and higher chemotherapy-related toxicities.

Obviously, this investigation had some limitations. First, the sample size for inclusion and completion of dynamic follow-up was modest. Despite the use of real-world nutritional management, patients with gastric cancer treated with XELOX/SOX chemotherapy are commonly treated with ambulatory chemotherapy modalities. This makes it difficult for them to receive adequate nutritional attention during the outpatient follow-up and results in ineffective outpatient nutritional management. In addition, patient exercise is also very important. In the pre-study, the research team did not focus on patient exercise education, which was one of the limitations of this study.

## 7 Conclusion

In conclusion, the results of this prospective clinical study confirm that patients with gastric cancer treated with XELOX/SOX chemotherapy experience a progressive reduction in skeletal muscle mass with each cycle of treatment. This reduction was accompanied by a higher occurrence of grade ≥ 3 neutropenia. In the future, it is recommended to incorporate patient-initiated reporting of clinical outcomes to enhance the effectiveness of interventions intended at reducing skeletal muscle quantity and mass during chemotherapy and mitigating the toxic side effects of chemotherapy. Furthermore, it should be organically combined with the body composition index in clinical practice to further optimize and individualize the traditional model of administering strength based on BSA.

## Data availability statement

The original contributions presented in the study are included in the article/supplementary material. Further inquiries can be directed to the corresponding authors.

## Ethics statement

The study was approved by Ethics Committee of the Ordos Central Hospital (No.2023-21). The studies were conducted in accordance with the local legislation and institutional requirements. The participants provided their written informed consent to participate in this study.

## Author contributions

Z-HL: Funding acquisition, Investigation, Project administration, Visualization, Writing – review & editing. TX: Data curation, Methodology, Validation, Visualization, Writing – review & editing. Y-JZ: Conceptualization, Data curation, Formal analysis, Investigation, Resources, Writing – review & editing. J-HJ: Data curation, Investigation, Methodology, Project administration, Writing – review & editing. Y-ZM: Data curation, Investigation, Methodology, Project administration, Validation, Writing – review & editing. J-XL: Conceptualization, Formal analysis, Methodology, Project administration, Writing – review & editing. JS: Writing – review & editing, Data curation, Formal analysis, Investigation, Project administration, Software, Visualization. Y-RF: Data curation, Investigation, Project administration, Validation, Writing – review & editing. B-YQ: Data curation, Investigation, Methodology, Resources, Writing – review & editing. FL: Data curation, Formal analysis, Investigation, Project administration, Writing – review & editing. D-JF: Data curation, Formal analysis, Investigation, Methodology, Project administration, Software, Writing – review & editing. M-JY: Data curation, Methodology, Software, Writing – review & editing. S-MM: Data curation, Formal analysis, Funding acquisition, Methodology, Resources, Validation, Writing – original draft, Writing – review & editing. Q-FL: Data curation, Funding acquisition, Methodology, Project administration, Supervision, Validation, Writing – original draft, Writing – review & editing.
